# Physiological effects of high-flow nasal cannula oxygen therapy after extubation: a randomized crossover study

**DOI:** 10.1186/s13613-023-01203-z

**Published:** 2023-10-18

**Authors:** Roque Basoalto, L. Felipe Damiani, Yorschua Jalil, María Consuelo Bachmann, Vanessa Oviedo, Leyla Alegría, Emilio Daniel Valenzuela, Maximiliano Rovegno, Pablo Ruiz-Rudolph, Rodrigo Cornejo, Jaime Retamal, Guillermo Bugedo, Arnaud W. Thille, Alejandro Bruhn

**Affiliations:** 1 Departamento de Medicina Intensiva, Facultad de Medicina, Pontificia Universidad Católica de Chile, Diagonal Paraguay 362, 6º Piso, P.O. Box 114D, 8330077 Santiago, Chile; 2 Programa de Medicina Física y Rehabilitación, Red Salud UC-CHRISTUS, Santiago, Chile; 3 CardioREspirAtory Research Laboratory (CREAR), Departamento de Ciencias de la Salud, Pontificia Universidad Católica de Chile, Santiago, Chile; 4 Departamento de Ciencias de la Salud, Facultad de Medicina, Pontificia Universidad Católica de Chile, Santiago, Chile; 5 Carrera de Kinesiología, Facultad de Medicina, Universidad de los Andes, Santiago, Chile; 6 Departamento de Salud del Adulto y Senescente, Escuela de. Enfermería, Pontificia Universidad Católica de Chile, Santiago, Chile; 7 Programa de Epidemiología, Instituto de Salud Poblacional, Facultad de Medicina, Universidad de Chile, Santiago, Chile; 8 Unidad de Pacientes Críticos, Departamento de Medicina, Hospital Clínico Universidad de Chile, Santiago, Chile; 9 Médecine Intensive Réanimation, Centre Hospitalier Universitaire de Poitiers, Poitiers, France; 10 INSERM Centre d’Investigation Clinique 1402 IS-ALIVE, Université de Poitiers, Poitiers, France; 11 Center of Acute Respiratory Critical Illness (ARCI), Santiago, Chile

**Keywords:** Weaning, Work of breathing, Esophageal pressure, Reintubation

## Abstract

**Background:**

Prophylactic high-flow nasal cannula (HFNC) oxygen therapy can decrease the risk of extubation failure. It is frequently used in the postextubation phase alone or in combination with noninvasive ventilation. However, its physiological effects in this setting have not been thoroughly investigated. The aim of this study was to determine comprehensively the effects of HFNC applied after extubation on respiratory effort, diaphragm activity, gas exchange, ventilation distribution, and cardiovascular biomarkers.

**Methods:**

This was a prospective randomized crossover physiological study in critically ill patients comparing 1 h of HFNC versus 1 h of standard oxygen after extubation. The main inclusion criteria were mechanical ventilation for at least 48 h due to acute respiratory failure, and extubation after a successful spontaneous breathing trial (SBT). We measured respiratory effort through esophageal/transdiaphragmatic pressures, and diaphragm electrical activity (ΔEAdi). Lung volumes and ventilation distribution were estimated by electrical impedance tomography. Arterial and central venous blood gases were analyzed, as well as cardiac stress biomarkers.

**Results:**

We enrolled 22 patients (age 59 ± 17 years; 9 women) who had been intubated for 8 ± 6 days before extubation. Respiratory effort was significantly lower with HFNC than with standard oxygen therapy, as evidenced by esophageal pressure swings (5.3 [4.2–7.1] vs. 7.2 [5.6–10.3] cmH_2_O; *p *< 0.001), pressure–time product (85 [67–140] vs. 156 [114–238] cmH_2_O*s/min; *p *< 0.001) and ΔEAdi (10 [7–13] vs. 14 [9–16] µV; *p *= 0.022). In addition, HFNC induced increases in end-expiratory lung volume and PaO_2_/FiO_2_ ratio, decreases in respiratory rate and ventilatory ratio, while no changes were observed in systemic hemodynamics, Troponin T, or in amino-terminal pro-B-type natriuretic peptide.

**Conclusions:**

Prophylactic application of HFNC after extubation provides substantial respiratory support and unloads respiratory muscles.

*Trial registration* January 15, 2021. NCT04711759.

**Supplementary Information:**

The online version contains supplementary material available at 10.1186/s13613-023-01203-z.

## Background

Postextubation respiratory failure and reintubation are frequent complications during weaning of mechanical ventilation in intensive care units (ICUs) [1, 2]. Reintubation occurs in approximately 15% of patients in ICU and has been associated with a worse prognosis so there is a strong interest in its prevention [3, 4]. High-flow nasal cannula (HFNC) oxygen therapy has been increasingly studied as a strategy to prevent extubation failure [5–8].

Several large clinical trials have evaluated the efficacy of postextubation HFNC on weaning outcomes [5–11]. Compared to standard oxygen, some trials have shown decreased reintubation rates [5, 7] while others have been unconclusive [10] or have shown no differences [8, 12]. The differing results of these trials may be explained by variable physiological effects of HFNC according to the clinical characteristics of the studied populations. Unfortunately, few studies have evaluated the physiological effects of HFNC after extubation [5, 13, 14]. Respiratory pump insufficiency and cardiovascular dysfunction have been described as the primary physiological mechanisms involved in extubation failure [15]. Maggiore et al. showed that prophylactic application of HFNC immediately after extubation improved oxygenation and decreased respiratory rate compared to standard oxygen, and decreased the risk of reintubation [5], but no studies have assessed whether HFNC modifies the pathophysiologic mechanisms leading to weaning failure: e.g., unbalance between work of breathing and patients’ capacity [16] or cardiovascular overload induced by weaning [17]. It may be relevant to quantify and characterize the support that HFNC is providing to the patient, as this may orient a more personalized approach to define when it should be used and when it may be weaned off.

The aim of the study was to compare comprehensively the effects of HFNC versus standard oxygen on respiratory effort, diaphragm activity, gas exchange, ventilation distribution, and cardiovascular biomarkers after extubation. Some of the results of this study have been previously presented in the form of an abstract [18].

## Methods

We conducted a prospective, randomized crossover physiological study between January 2021 and December 2022 in the ICU of the Hospital Clínico UC-CHRISTUS, Santiago, Chile. The study was approved by the ethical committee of the Pontificia Universidad Católica de Chile (N° 180,814,001), and it was registered in clinicalTrials.gov (NCT04711759).

All patients intubated for acute respiratory failure and mechanically ventilated for at least 48 h, in whom a spontaneous breathing trial (SBT) was planned by the attending physician, were screened for eligibility. Exclusion criteria were age < 18 years old, intubation for acute exacerbation of chronic obstructive pulmonary disease, contraindications to place an esophageal balloon catheter (e.g., coagulopathy, esophageal varices, recent gastric surgery), or for the use of electrical impedance tomography (EIT) (e.g., pacemaker), tracheostomy, or do not reintubate order after extubation. If patients were eligible, informed consent was obtained from the next of kin, and before performing the SBT, the study monitoring was placed. All patients underwent a 30-min SBT with positive end expiratory pressure (PEEP) 0 cmH_2_O and pressure support of 8 cmH_2_O. Inclusion in the study was confirmed and randomization performed only for patients who passed successfully the SBT, once they were extubated.

### Study protocol

After extubation, patients were assigned to one of two sequences in a random order performed by a computer using the R Statistical software with blocks of 10. Patients allocated to the first sequence received standard oxygen therapy through a venturi mask for1 h (ACU-FLOW, REUTTER, Chile; the flow of oxygen was adjusted according to the FiO2), followed by 1 h of HFNC (AIRVO 2, Fisher and Paykel Healthcare, Auckland, New Zealand) with a gas flow of 50 L/min. Patients allocated to the second sequence received HFNC for 1 h, followed by 1 h of standard oxygen therapy through a venturi mask. Inspired O2 fraction was kept constant throughout the protocol. A washout period was not included as the effects of HFNC and standard oxygen were assumed to dissipate rapidly after stopping them, so it was considered unlikely that the effects of the first intervention would extend until the end of the second period. Other similar studies have used the same approach [19]. At the end of each phase of the study, blood samples were collected for arterial and central venous gases, ultrasensitive troponin T, amino-terminal pro-B-type natriuretic peptide (NT-ProBNP), and hemodynamic and respiratory variables were registered.

### Physiological recordings

All patients were positioned at 45° and were ventilated with a Servo-i ventilator (Maquet Critical Care, Solna, Sweden). A nasogastric catheter with electrodes to record the electric activity of the diaphragm (EAdi), and with esophageal and gastric balloons (Neurovent Research Inc., Toronto, Canada) was installed before the SBT. The placement of the catheter was guided by the EAdi signal and the correct position was confirmed by an occlusion test as previously described [20–22]. The esophageal pressure (Pes) signal was prioritized to define the optimal position in case of disagreement between EAdi signal and Pes. Esophageal and gastric pressures (Pga) were recorded through an analog/digital interface converter (BIOPAC^®^ Systems, Inc). The EAdi signal was collected from the RS232 port of SERVO-i ventilator at a sampling rate of 100 Hz (servo tracker software, Maquet Getinge group Critical Care, Solna, Sweden) and recorded in a laptop.

An electrical impedance tomography belt with 32 electrodes (Enlight 1800, Timpel, Brazil) was placed around the patient´s thorax at the fifth intercostal space. Before extubation, we connected the EIT flow sensor to the endotracheal tube and recorded 20 spontaneous breaths without positive pressure to calibrate the EIT signal (ΔZ) against measured tidal volumes, in order to estimate changes in lung volumes after extubation [23].

Waveforms from EAdi, EIT, esophageal and gastric pressures were recorded during the last 5 min of each phase and synchronized offline (Additional file 1: Fig. S1). One minute of stable signal was analyzed in each recording. All signals were analyzed with AcqKnowledge software (Biopac Systems, Santa Barbara, CA).

### Measurement of physiological variables

Based on acquired signals from the esophageal/gastric pressure, EIT and EAdi monitoring, the following variables were calculated offline blinded to the sequence allocation:

- Esophageal pressure swing (ΔPes): The average variation of esophageal pressure during inspiration, calculated as the difference between end-expiratory and end-inspiratory esophageal pressure.
- Pressure–time product per breath (PTP): The average of the area subtended by the Pes curve from the onset to the end of inspiratory effort by simplified calculation [24].
- Pressure–time product per minute (PTPmin): The average of the sum of PTP over one minute.
- Change in transdiaphragmatic pressure (ΔPdi): Transdiaphragmatic pressure (Pdi) was derived by subtracting Pes from Pga., while its change was calculated as the average variation of Pdi during inspiration [25].
- Global and regional tidal volume (V_T_): The tidal variation of impedance (ΔZ) was analyzed globally and divided into two regions of interest (dependent and non-dependent). Based on the previous calibration we estimated V_T_ (V_T-Glob_) and subdivided it in a dependent and non-dependent V_T_ (V_T-Dep,_ and V_T-Nondep_).
- Minute ventilation: We estimated minute ventilation ($\dot{\mathrm{V}}{\mathrm{E }}_{\mathrm{estimated}}$) as the product of respiratory rate x V_T-Glob_.
- Ventilatory ratio (VR): Index of impaired efficiency of ventilation calculated by the following Equation [26]:
  $$\mathrm{VR}=\frac{\dot{\mathrm{V}}{\mathrm{E }}_{\mathrm{estimated}} \times {\mathrm{PaCO}}_{2\,\mathrm{ measured}}}{\dot{\mathrm{V}}{\mathrm{E }}_{\mathrm{predicted}} \times {\mathrm{PaCO}}_{2\,\mathrm{ predicted}}}.$$
- Variation of end-expiratory lung volume (ΔEELV): We calculated the difference in end-expiratory lung impedance (EELI) between standard oxygen and HFNC and, based on the previous calibration, we transformed this impedance difference into ΔEELV.
- Global inhomogeneity index (GI): Calculated as the sum of the impedance changes of each pixel concerning its median (in absolute values), divided by the sum of the impedance values of each pixel [27, 28].
- Anteroposterior ventilation ratio (A/P ratio): Calculated as the ratio between the sum of tidal impedance changes in the anterior and posterior halves of the functional image or the lung region of interest within it [29].
- Center of ventilation (COV): We determined it as a vertical coordinate that marks the point where the sum of the regional ventilation (ventral and dorsal) divides the lung into two equal parts [27].
- Electrical activity of the diaphragm (ΔEAdi): Defined as the amplitude of EAdi during inspiration.
- Diaphragmatic neuromuscular coupling: Calculated as the ratio between ΔPdi and ΔEAdi (ΔPdi/ΔEadi) [30].
- Neuroventilatory efficiency (NVE): Calculated as the ratio of V_T-Glob_ to ΔEAdi (V_T-glob_/ΔEAdi) [31].
- Dynamic compliance (Cdyn): (V_T-glob_/ΔPes): Calculated as the ratio of V_T-Glob_ to ΔPes (V_T-Glob_/ΔPes) [19].

### Statistical analysis

Based on an alpha value of 0.05, power set at 80%, and assuming a similar effect size to that previously reported in a study performed in acute hypoxemic respiratory failure patients [19], which found that HFNC induced a decrease of 61 ± 95 cmH2O·s/min in pressure–time product per minute, compared to standard oxygen, we calculated that a sample size of 22 subjects would be required.

Demographic data for all subjects were summarized using descriptive statistics. The Shapiro–Wilk normality test was used to verify data distribution normality. Data are represented as mean ± standard deviation (SD) or median and interquartile range, as appropriate. We followed recommendations for crossover trials to compare the effects of oxygen mask versus HFNC [32].

To analyze the effect of treatment, sequence, and carryover effect, we applied two-way repeated measures ANOVA. Linear regression was applied to analyze whether clinical variables could predict the magnitude of change in PTPmin induced by HFNC. GraphPad Prism v7 (GraphPad Software, San Diego, CA, USA) was used for calculations.

## Results

Twenty-five patients were initially recruited in the study, but as 3 of them failed the SBT, only 22 were finally enrolled. Nine were women (41%), with a mean age of 59 ± 17 years old, a Charlson comorbidity index of 3 ± 2. APACHE II at ICU admission was 14 ± 8, and SOFA score the day of the study was 5 ± 3. Patients had been intubated for 8 ± 6 days before extubation. The main etiologies of acute respiratory failure were SARS-CoV-2 (*n = *14, 63%), aspiration pneumonia (*n = *3, 14%), bacterial pneumonia (*n = *2, 9%), and other respiratory viruses (*n = *2, 9%). The main characteristics of patients are reported in Table 1. Eleven patients were allocated to each sequence without differences between groups at baseline (Additional file 1: Table S1). All patients completed the study protocol, and no adverse events were observed. No significant change was observed for any of the study variables when comparing the first and the second study periods, irrespective of the sequence allocation (Additional file 1: Table S2).

**Table 1:** Baseline patient characteristics

Characteristics	All patients (*n = *22)
Age, years	59 ± 17
Female sex, n %	9 (41)
Body mass index, kg/mt^2^	32 ± 9
Charlson comorbidity index	3 ± 2
APACHE II at ICU admission	14 ± 8
SOFA (day of the study)	5 ± 3
SOFA (peak during ICU)	7 ± 3
PaO_2_/FiO_2_ (lowest value during MV), mmHg	136 ± 56
Length of mechanical ventilation, days	8 ± 4
Characteristics at the end of the SBT	
Heart rate, bpm	90 ± 12
Systolic arterial pressure, mmHg	145 ± 23
Diastolic arterial pressure, mmHg	99 ± 16
Mean arterial pressure, mmHg	76 ± 18
Respiratory rate, bpm	22 ± 5
Tidal volume, ml	479 ± 133
P 0.1, cmH_2_O	0.77 ± 0.99
PaO_2_/FiO_2_	242 ± 67
PaCO_2_, mmHg	36 ± 5
pH	7.46 ± 0.05

Abbreviations: APACHE: Acute Physiology and Chronic Health Evaluation; SOFA: Sequential Organ Failure Assessment; ICU: intensive care unit; AHRF: acute hypoxemic respiratory failure; MV: mechanical ventilation; SBT: spontaneous breathing trial; P 0.1: airway occlusion pressure at 100 ms; PaCO_2_: partial pressure of carbon dioxide. Variables are expressed as the mean ± standard deviation

### Respiratory effort

**Fig. 1:**
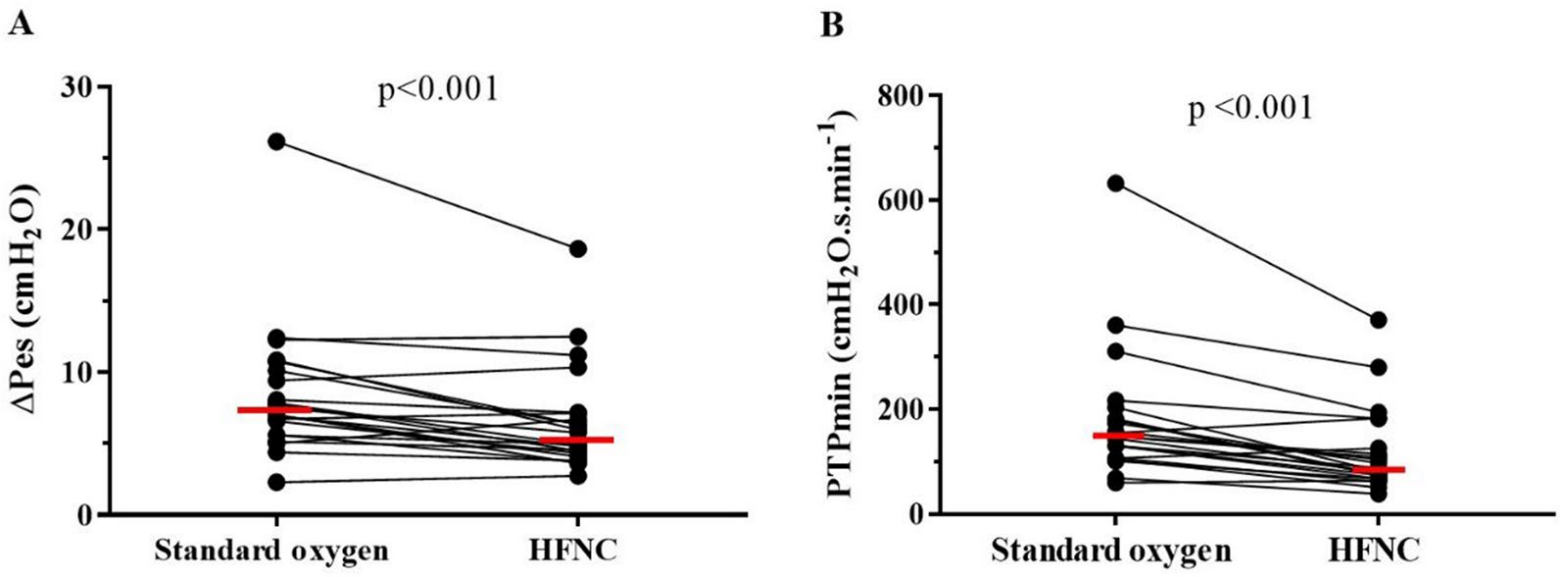
High-flow nasal cannula (HFNC) oxygen therapy applied after extubation decreases work of breathing and respiratory rate. HFNC reduces inspiratory esophageal pressure swings (**A**) and pressure–time product per minute (**B**) in comparison with standard oxygen This indicates a lower metabolic cost and respiratory effort associated with the use of HFNC. Horizontal red lines represent median values. *P* values were calculated using a two-way repeated measures ANOVA

The respiratory effort was significantly lower with HFNC than with standard oxygen therapy, evidenced by a median decrease of 26% (interquartile range (IQR) 7–38%) in ΔPes, 35% (IQR 15–41%) in PTP per breath, and 39% (IQR 22–45%) in PTPmin (Table 2, Fig. 1). ΔPdi was also significantly lower with HFNC than with standard oxygen (Table 2). Dynamic compliance evaluated by V_T_/ΔPes was higher with HFNC than with standard oxygen therapy.

**Table 2:** Effects on high-flow nasal cannula versus standard oxygen on respiratory variables

Variable	Standard oxygen	HFNC	*p* value
ΔPes, cmH_2_O	7.2 [5.6–10.3]	5.3 [4.2–7.1]	< 0.001
PTP, cmH_2_O. s	6.4 [5.3–8.9]	4.3 ± [3.4–6.2]	< 0.001
PTP_min_, cmH_2_O. s/min	156 [114–238]	85 [67–140]	< 0.001
Pdi, cmH_2_O	7.3 [6.2–11.2]	5.9 [5.0–7.7]	0.008
Respiratory rate, bpm	24 ± 6	22 ± 5	0.001
V_T Glob_, ml	362 ± 112	334 ± 99	0.292
V_T Dep_, ml	261 ± 97	239 ± 76	0.206
V_T Non-Dep_, ml	101 ± 59	94 ± 60	0.554
V_T_, ml/kg IBW	6.2 ± 2.1	5.7 ± 1.9	0.482
Minute Ventilation, L/min	8.6 ± 2.8	7.4 ± 2.4	0.037
ΔEELV_glob_ (change from standard oxygen), ml	0 [0–0]	169 [93–656]	< 0.001
Dynamic compliance, V_T_/ΔPes (ml/cmH_2_0)	48 [33–62]	66 [40–82]	0.048
ΔEAdi, µV	14 [9–16]	10 [7–13]	0.022
Neuroventilatory efficiency, (V_T_/EAdi) ml/ µV	30 ± [24–36]	33 [22–54]	0.111
Diaphragmatic neuromuscular coupling, (Pdi/ΔEAdi) cmH2O/µV	0.65 [0.42–0.70]	0.61 [0.56–0.76]	0.256
A/P Ratio	0.41[0.25–0.62]	0.41 [0.16–0.59]	0.125
Center of ventilation	55 [52–58]	56 [50–60]	0.967
GI index	0.97 [0.87–1.38]	0.88 [0.75–1.5]	0.609
PaO_2_/FiO_2_, mmHg	240 ± 77	267 ± 80	0.012
PaCO_2_, mmHg	37 ± 4.7	37 ± 4.1	0.888
pH	7.45 ± 0.04	7.45 ± 0.05	0.898
Ventilatory ratio	1.36 ± 0.36	1.18 ± 0.39	0.035

Abbreviations: ΔPes: inspiratory esophageal pressure swing; PTP: pressure–time product per breath; PTP_min_: pressure–time product per minute; Pdi: transdiaphragmatic pressure; V_T Glob_: tidal volume global; V_T Dep_: tidal volume dependent region; V_T-Nondep_: tidal volume non-dependent region; ΔEELV_glob_: variation of end expiratory lung volume; ΔEAdi: electrical activity of the diaphragm; A/P ratio: anterior to posterior ventilation ratio; GI index: global inhomogeneity index; PaCO_2_: partial pressure of carbon dioxide. Normally distributed variables are expressed as the mean ± standard deviation; non-normal distributed variables are expressed as median and [interquartile range]. *p* values were calculated using a two-way repeated measures ANOVA

Regarding ΔEAdi, HFNC induced a median decrease of 17% (IQR 9–28%) compared to standard oxygen therapy. However, no differences were observed in diaphragmatic neuromuscular coupling or in neuroventilatory efficiency (Table 2).

None of the following clinical variables could predict the magnitude of change in PTPmin induced by HFNC: respiratory rate, minute ventilation or PaCO_2_, evaluated while the patient was on standard oxygen, or their change in response to HFNC. The only variable that predicted a larger impact of HFNC on PTPmin was a higher value of PTPmin while the patient was on standard oxygen indicating that the higher the PTPmin under standard oxygen, the greater the reduction of PTPmin under HFNC (Additional file 1: Table S3 and Fig. S2).

### Ventilation distribution

**Fig. 2:**
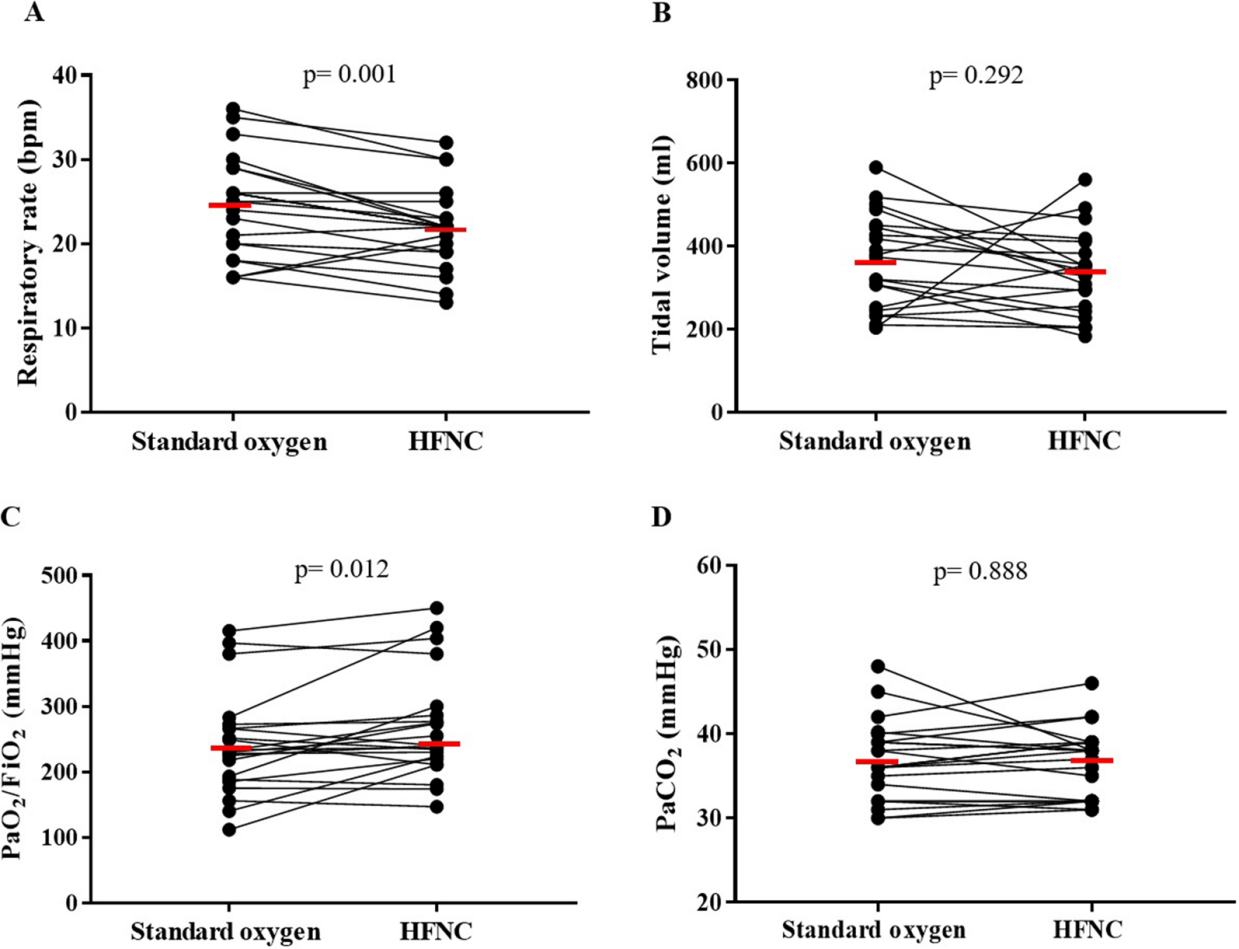
High-flow nasal cannula (HFNC) oxygen therapy applied after extubation decreases respiratory rate (**A**), has no effect on tidal volume (**B**), it increases PaO_2_/FiO_2_ ratio (**C**), and it did not modify arterial carbon dioxide levels (**D**), compared to standard oxygen. Horizontal red lines represent mean values. P values were calculated using a two-way repeated measures ANOVA

No differences were observed in the estimated V_T_ (V_T_ global) when comparing HFNC with standard oxygen therapy. However, due to a lower respiratory rate (22 ± 5 vs. 24 ± 6 bpm; *p =* 0.001), a median decrease of 16% (IQR 3–18%) in the estimated minute ventilation was observed with HFNC compared to standard oxygen therapy (Table 2, Fig. 2). EELV significantly increased with HFNC relative to standard oxygen therapy in a magnitude estimated in 169 [93–656] ml (*p *< 0.001). On the other hand, the distribution of ventilation was mainly dorsal without differences between HFNC and standard oxygen, evaluated either by A/P ratio or by COV (Table 2). Regarding GI index, no differences were found (Table 2).

### Gas exchange

HFNC slightly but consistently increased oxygenation, evaluated by PaO_2_/FiO_2_ ratio, with a median increment of 27 mmHg compared to standard oxygen (*p *= 0.012). The change in PaO_2_/FiO_2_ ratio in response to HFNC was not associated to ΔEELV (*R*^2^ = 0.002, *p *= 0.82). No differences were observed in PaCO_2_ (Fig. 2), but the ventilatory ratio was lower with HFNC (Table 2).

### Hemodynamics and cardiovascular stress biomarkers

NT-ProBNP did not vary between HFNC and standard oxygen therapy. Troponin T was only detected in 16 of the 22 patients and no differences were observed between HFNC and standard oxygen. In addition, no differences were found in any of the hemodynamic or perfusion variables evaluated (Table 3).

**Table 3:** Effects on high-flow nasal cannula versus standard oxygen on hemodynamics and cardiac biomarkers

Variable	Standard oxygen	HFNC	*p* value
Systolic arterial pressure, mmHg	143 ± 19	140 ± 19	0.238
Diastolic arterial pressure, mmHg	73 ± 15	71 ± 14	0.427
Mean arterial pressure, mmHg	98 ± 14	96 ± 13	0.310
Heart rate, bpm	85 ± 12	86 ± 12	0.238
CVP, mmHg	3 [1–7]	4 [1–6]	0.792
SvcO_2_, %	75 ± 10	75 ± 7	0.750
Pv-aCO_2_ gap	2.5 ± 1.9	3.4 ± 2.2	0.273
NT-proBNP, pg/ml	355 [152–901]	329 [147–962]	0.314
Troponin T, pg/ml	17.2 [10.5–29.9]	19.1 [10.3–32.0]	0.206

Abbreviations: HFNC: high-flow nasal cannula; SvcO_2_: central venous oxygen saturation; CVP: central venous pressure; Pv-aCO_2_ gap: venous-to-arterial carbon dioxide gap; NT-proBNP: N-terminal pro-B-type natriuretic peptide. Normally distributed variables are expressed as mean ± standard deviation; non-normal distributed variables are expressed as median and [interquartile range]. *p* values were calculated using a two-way repeated measures ANOVA

## Discussion

In the present randomized crossover study, we evaluated the physiological effects of HFNC compared to standard oxygen, during the early postextubation phase, in 22 ICU patients who had been ventilated after an episode of acute respiratory failure. We found that HFNC, compared to standard oxygen therapy, markedly decreased work of breathing, improved oxygenation and ventilatory efficiency, and increased end-expiratory lung volume, but no relevant impact on hemodynamics or cardiovascular stress biomarkers was observed. The present study presents the most complete analysis reported up to now of the physiological effects of HFNC during the postextubation phase.

The decrease in PTPmin induced by HFNC was mainly due to a lower PTP by breath, while the slight changes in respiratory rate contributed to a less extent. These effects are similar to those described by Mauri et al. in acute hypoxemic respiratory failure patients treated with HFNC to prevent intubation, at the early phase of the disease [19, 23]. HFNC also induced a decrease in transdiaphragmatic pressures and in EAdI, indicating marked unloading of the diaphragm. Interestingly, despite the lower respiratory effort, tidal volume remained unchanged. This observation has been previously reported by other authors [19, 33] and has been related to a decrease in inspiratory resistance [33–35]. The large magnitude of change in breathing effort observed in the present study, indicates that HFNC provides substantial respiratory support in the postextubation phase. Thus, the effectiveness of HFNC to prevent postextubation respiratory failure and reintubation, shown in previous clinical trials [5, 7], may be related to its ability to unload the respiratory muscles during inspiration.

In the study of Mauri et al. mentioned above, performed in patients with acute respiratory failure, the efficacy of HFNC to decrease respiratory effort was shown to be positively correlated to baseline PaCO_2_ [19]. In the present study, we did not observe that association. The individual impact of HFNC on PTPmin (difference compared to standard oxygen) was only correlated to the absolute PTPmin while on standard oxygen (Additional file 1: Table S3 and Figure S2), but not to any of the clinical variables analyzed. Therefore, the support provided by HFNC in the postextubation phase, which can exceed 50% of the breathing effort, may be not evident by usual clinical monitoring. This should be considered when weaning HFNC during the postextubation phase. In a previous clinical trial in which HFNC was stopped by protocol 24 h after extubation [6], a sudden increase in reintubation rates was observed after switching to conventional oxygen.

Ventilatory ratio decreased confirming the well-known effect of HFNC on dead space [19, 23, 36, 37]. However, the decrease in minute ventilation observed with HFNC was lower than reported in previous studies of HFNC during acute respiratory failure [19], and we observed no relation with the change in PTPmin.

HFNC induced a mild improvement in oxygenation. This effect has been related to a positive airway pressure effect and prevention of atelectasis [23, 38, 39]. In fact, we observed an increased EELV with HFNC, which we estimated in 169 ml as median value, suggesting a slightly higher functional residual capacity. However, patients were not instructed to keep their mouths closed while breathing with HFNC so the PEEP effect may have been attenuated [40]. It has been shown that the impact of HFNC on EELV may be proportional to the flow rate applied, which in the present study was 50 LPM [23]. Interestingly, the gas flow delivered with the venturi mask was also close to 50 LPM, but the effects of high flow are completely different when it is delivered directly into the nostrils compared to delivering it through a venturi mask.

In contrast to a previous study in patients with acute hypoxemic respiratory failure at risk of intubation, which observed a decrease in inhomogeneity with HFNC [19], we did not observe such effect. However, the global inhomogeneity index values in the present study reflected that the distribution of ventilation was already quite homogeneous. We also did not observe any effect of HFNC on the distribution of ventilation between anterior and posterior lung regions.

As cardiac dysfunction is a common cause of weaning failure, and because HFNC may theoretically influence cardiovascular function, we included several hemodynamic variables and cardiovascular biomarkers in the study. We observed no influence of HFNC on any of these variables. NT-Pro-BNP was elevated above 1000 pg/ml in 4 patients but even in this subgroup no evidence of an effect of HFNC was observed. This data indicates that HFNC does not have relevant acute hemodynamic effects in the postextubation phase, even though we showed that it decreases negative swings in intrathoracic pressure, and that a certain CPAP effect has been previously reported [40].

The present study has some limitations. The study period was limited to the first hours after extubation, with a rather short period of observation for each intervention. Although most of the respiratory effects of HFNC are expected to be rapid, some of the potential cardiovascular effects may occur with a slower dynamic and may have been missed. Second, although changes in impedance values obtained with EIT have been used to estimate changes in lung volumes, this approach may be inaccurate due to limitations of the technique, such as the limited transversal lung region analyzed with conventional EIT belts. Third, HFNC was applied at a fix flow of 50 lpm, which may not be the optimal flow for each individual patient. However, there is no clear criteria to titrate flow with HFNC and using different flows may have introduced an additional source of variability.

## Conclusions

We conclude that prophylactic application of HFNC after extubation, in patients previously intubated for acute respiratory failure, provides substantial respiratory support and respiratory muscle unloading, evidenced by a relevant decrease in respiratory effort, and mild improvements in oxygenation, ventilatory efficiency and end-expiratory lung volume. Although HFNC induces a slight decrease in respiratory rate, this effect does not appear to be a good indicator of the large reduction in energy consumption by the respiratory muscles. HFNC does not impact hemodynamics or cardiovascular stress markers. Decreased respiratory effort may explain the effectiveness of HFNC to prevent extubation failure.

### Supplementary Information

**Additional file 1: Table S1.** Baseline patient characteristics by sequence. **Table S2.** Respiratory and hemodynamics variables per period. **Table S3**. Variables associated to the individual effect of high flow nasal cannula (relative to standard oxygen) on pressure time product per minute. **Figure S1.** Offline synchronization of physiological waveforms in a representative patient during with standard oxygen and with high-flow nasal cannula oxygen therapy. Vertical dotted lines indicate the start of inspiration. Pes: Esophageal pressure; EAdi: Electrical activity of the diaphragm; Pdi: Transdiaphragmatic pressure; ΔZ: Global impedance variation. **Figure S2.** Pressure time product per minute during standard oxygen is correlated with the relative decrease in the same variable induced by high flow nasal cannula.

## Data Availability

The datasets used and/or analyzed during the current study are available from the corresponding author on reasonable request.

## Abbreviations

HFNC: High-flow nasal cannula
SBT: Spontaneous breathing trial
EAdi: Electrical activity of the diaphragm
EIT: Electrical impedance tomography
ICU: Intensive care unit
PEEP: Positive end expiratory pressure
L/min: Liters per minute
NT-ProBNP: Amino-terminal pro-B-type natriuretic peptide
cmH_2_O: Centimeters of water
ΔPes: Esophageal pressure swing
PTP: Pressure–time product per breath
PTP: Pressure–time product per minute
Pdi: Transdiaphragmatic pressure
Pga: Gastric pressure
V_T_: Tidal volume
V_T-Glob_: Tidal volume global (estimated from EIT)
V_T-Dep_: Tidal volume dependent region (estimated from EIT)
V_T-Nondep_: Tidal volume non-dependent region (estimated from EIT)
ΔZ: Tidal variation of impedance
VR: Ventilatory ratio
PaCO_2_: Partial pressure of carbon dioxide
EELV: End-expiratory lung volume
GI: Global inhomogeneity index
A/P ratio: Anteroposterior ventilation ratio
COV: Center of ventilation
NVE: Neuroventilatory efficiency
Cdyn: Dynamic compliance
APACHE II: Acute Physiology and Chronic Health disease Classification System II
SOFA: Sepsis related Organ Failure Assessment
