# Supplementary material for: Physiological effects of high-flow nasal cannula oxygen therapy after extubation: a randomized crossover study
Source: Ann Intensive Care. 2023 Oct 18;13:104. doi: 10.1186/s13613-023-01203-z (PMC10584771; doi:10.1186/s13613-023-01203-z)
Supplement: Supplementary file 1 — Additional file 1: Table S1. Baseline patient characteristics by sequence. Table S2. Respiratory and hemodynamics variables per period. Table S3. Variables associated to the individual effect of high flow nasal cannula (relative to standard oxygen) on pressure time product per minute. Figure S1. Offline synchronization of physiological waveforms in a representative patient during with standard oxygen and with high-flow nasal cannula oxygen therapy. Vertical dotted lines indicate the start of inspiration. Pes: Esophageal pressure; EAdi: Electrical activity of the diaphragm; Pdi: Transdiaphragmatic pressure; ΔZ: Global impedance variation. Figure S2. Pressure time product per minute during standard oxygen is correlated with the relative decrease in the same variable induced by high flow nasal cannula. [file 13613_2023_1203_MOESM1_ESM.docx]

**Additional file 1 (supplementary information)**

**TITLE: PHYSIOLOGICAL EFFECTS OF HIGH-FLOW NASAL CANNULA OXYGEN THERAPY AFTER EXTUBATION: A RANDOMIZED CROSSOVER STUDY**

Roque Basoalto; L. Felipe Damiani; Yorschua Jalil; Maria Consuelo Bachmann; Vanessa Oviedo; Leyla Alegría; Emilio Daniel Valenzuela; Maximiliano Rovegno; Pablo Ruiz-Rudolph; Rodrigo Cornejo; Jaime Retamal; Guillermo Bugedo; Arnaud W. Thille; Alejandro Bruhn

**Table S1.** Baseline patient characteristics by sequence

|  | **Standard oxygen-HFNC (n= 11)** | **HFNC-**  **Standard oxygen**  **(n= 11)** | **P Value** |
| --- | --- | --- | --- |
| **Age, years** | 60 ± 14 | 57 ± 21 | 0.6104 |
| **Female, n** | 4 | 5 | - |
| **Body mass index, kg/m^2^** | 32.3 [28.3 – 38.8] | 31.2 [23.1 – 32.8] | 0.1566 |
| **Charlson comorbidity index** | 3 ± 3 | 3 ± 2 | 0.8016 |
| **APACHE II admission** | 15 ± 10 | 14 ± 6 | 0.7340 |
| **PaO_2_/FiO_2_ (lowest value during MV), mmHg** | 128 ± 50 | 147 ± 63 | 0.4273 |
| **Length of mechanical ventilation, d** | 8 ± 4 | 7 ± 4 | 0.6582 |
| **Characteristics at the end of the SBT** |  |  |  |
| **Heart rate, bpm** | 87 ± 16 | 89 ± 9 | 0.7399 |
| **Systolic arterial pressure, mmHg** | 140 ± 24 | 150 ± 23 | 0.2773 |
| **Diastolic arterial pressure, mmHg** | 77 ±20 | 75 ± 16 | 0.7932 |
| **Mean arterial pressure, mmHg** | 97 ± 15 | 102 ± 17 | 0.4528 |
| **Respiratory rate, bpm** | 22 ± 6 | 22 ± 3 | 0.8748 |
| **Minute Ventilation, L/min** | 10.5 ± 1.3 | 9.6 ± 1.9 | 0.2170 |
| **P 0.1, cmH_2_O** | 0.6 [0.5 – 1.8] | 0.4 [0.3 0.6] | 0.0762 |
| **PaO_2_/FiO_2_, mmHg** | 211 [195 – 241] | 250 [209 - 360] | 0.0719 |
| **pH** | 7.47 ± 0.06 | 7.45 ± 0.04 | 0.3447 |
| **PaCO_2_, mmHg** | 36 ± 4 | 34 ± 5 | 0.3037 |

Abbreviations: APACHE: Acute Physiology and Chronic Health Evaluation; SOFA: Sequential Organ Failure Assessment; ICU: Intensive care unit; MV: Mechanical ventilation; SBT: Spontaneous breathing trial; P 0.1: airway occlusion pressure at 100 milliseconds; PaCO_2_: Partial pressure of carbon dioxide. Normally distributed variables are expressed as the mean ± standard deviation; non-normal distributed variables are expressed as median and [interquartile range]. *P value< 0.005 by *t*-test or Mann-Whitney test, as appropriate.

**Table S2.** Respiratory and hemodynamics variables per period

| **Variable** | **Time 1** | **Time 2** | **P Value** |
| --- | --- | --- | --- |
| **ΔPes, cmH_2_O** | 6.5 [4.8 – 8.4] | 6.7 [4.8 – 10.4] | 0.823 |
| **PTPmin, cmH_2_O. s/min** | 118 [79 - 175] | 123 [74 - 183] | 0.502 |
| **Pdi, cmH_2_O** | 7.2 [5.6 - 9.1] | 6.3 [5.5 – 8.9] | 0.328 |
| **Respiratory rate, bpm** | 23 ± 5 | 24 ± 6 | 0.536 |
| **Tidal Volume, ml** | 339 ± 109 | 356 ± 104 | 0.524 |
| **Minute ventilation, L/min** | 7.7 ± 2.6 | 8.2 ± 2.6 | 0.351 |
| **ΔEELI_glob_, AU** | -1.29 [-23.68 – 20.74] | 4.31 [-13.99 – 35.04] | 0.5624 |
| **Dynamic compliance, V_T_/ΔPes (ml/cmH_2_0)** | 56 [34 - 73] | 57 [35 - 81] | 0.373 |
| **ΔEAdi, µV** | 13 [9 – 15] | 10 [8 – 15] | 0.4332 |
| **PaO_2_/FiO_2_, mmHg** | 257 ± 85 | 252 ± 74 | 0.693 |
| **PaCO_2_, mmHg** | 37 ± 4.8 | 36.6 ± 4.0 | 0.885 |
| **pH** | 7.45 ± 0.05 | 7.45 ± 0.04 | 0.951 |
| **Mean arterial pressure, mmHg** | 98 ± 16 | 96 ± 12 | 0.386 |
| **Heart rate, bpm** | 85 ± 12 | 86 ± 12 | 0.590 |
| **SvcO_2_, %** | 76 ± 6 | 74 ± 10 | 0.333 |
| **Pv-aCO_2_ gap, mmHg** | 2.9 ± 1.9 | 3.0 ± 2.3 | 0.919 |
| **NT-proBNP, pg/ml** | 336 [146 - 901] | 343 [153 - 962] | 0.340 |
| **Troponin T, pg/ml** | 16.3 [11.8 – 26-58] | 17.7 [10.3 – 28.4] | 0.117 |

Abbreviations: ΔPes: Inspiratory esophageal pressure swing; PTP: Pressure time product per breath; PTP_min_: Pressure time product per minute; Pdi: Transdiaphragmatic pressure; EELI: End expiratory lung impedance; AU: Arbitrary units; ΔEAdi: Electrical activity of the diaphragm; PaCO_2_: Partial pressure of carbon dioxide; SvcO_2_: Central venous oxygen saturation; Pv-aCO_2_ gap: Venous-to-arterial carbon dioxide gap; NT-proBNP: N-terminal pro-B-type natriuretic peptide. Normally distributed variables are expressed as the mean ± standard deviation; non-normal distributed variables are expressed as median and [interquartile range]. *P value < 0.05 by paired t test or by Wilcoxon signed rank test as appropriate.

**Table S3**. Variables associated to the individual effect of high flow nasal cannula (relative to standard oxygen) on pressure time product per minute.

|  | **R^2^** | **P value** |
| --- | --- | --- |
| **Respiratory rate with standard oxygen** | 0.0091 | 0.6727 |
| **Minute ventilation with standard oxygen** | 0.0017 | 0.8613 |
| **PaCO_2_ with standard oxygen** | 0.0007 | 0.9063 |
| **PTPmin with standard oxygen** | 0.6902 | **˂0.0001** |
| **Δ Respiratory rate (HFNC – SO)** | 0.0379 | 0.3730 |
| **Δ Minute ventilation (HFNC – SO)** | 0.0755 | 0.2156 |
| **Δ PaCO_2_ (HFNC – SO)** | 0.0031 | 0.8055 |

Abbreviations: PTPmin: Pressure time product per minute; PaCO_2_: Partial pressure of carbon dioxide; HFNC – SO: Represents the difference between High flow nasal cannula and Standard oxygen.


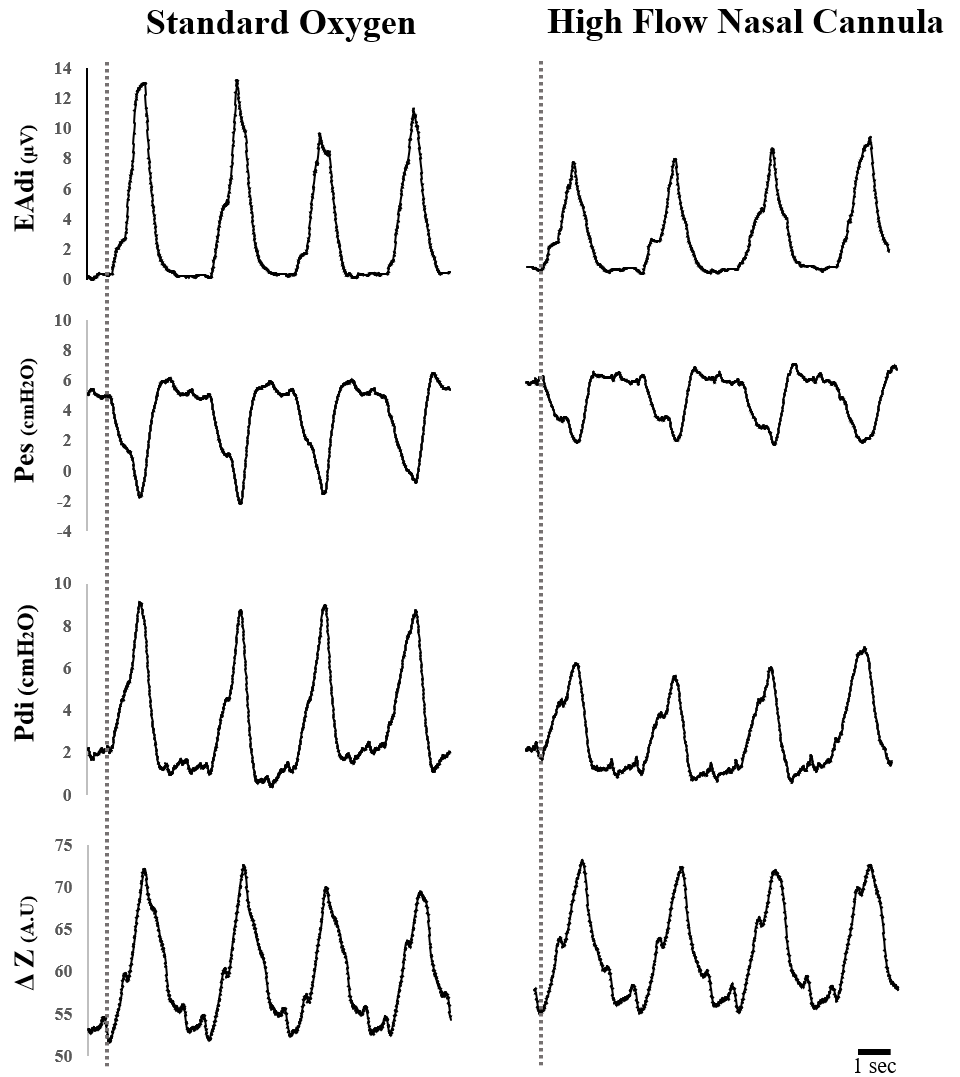


**Figure S1.** Offline synchronization of physiological waveforms in a representative patient during with standard oxygen and with high-flow nasal cannula oxygen therapy. Vertical dotted lines indicate the start of inspiration. Pes: Esophageal pressure; EAdi: Electrical activity of the diaphragm; Pdi: Transdiaphragmatic pressure; ΔZ: Global impedance variation.

**Figure S2.** Pressure time product per minute during standard oxygen is correlated with the relative decrease in the same variable induced by high flow nasal cannula.
